# Effect of neural network structure in accelerating performance and accuracy of a convolutional neural network with GPU/TPU for image analytics

**DOI:** 10.7717/peerj-cs.909

**Published:** 2022-03-03

**Authors:** Aswathy Ravikumar, Harini Sriraman, P. Maruthi Sai Saketh, Saddikuti Lokesh, Abhiram Karanam

**Affiliations:** School of Computer Science and Engineering, Vellore Institute of Technology, Chennai, Tamil Nadu, India

**Keywords:** Deep convolutional neural networks, Acceleration, Image processing, High-performance computing, Image analytics

## Abstract

**Background:**

In deep learning the most significant breakthrough in the field of image recognition, object detection language processing was done by Convolutional Neural Network (CNN). Rapid growth in data and neural networks the performance of the DNN algorithms depends on the computation power and the storage capacity of the devices.

**Methods:**

In this paper, the convolutional neural network used for various image applications was studied and its acceleration in the various platforms like CPU, GPU, TPU was done. The neural network structure and the computing power and characteristics of the GPU, TPU was analyzed and summarized, the effect of these on accelerating the tasks is also explained. Cross-platform comparison of the CNN was done using three image applications the face mask detection (object detection/Computer Vision), Virus Detection in Plants (Image Classification: agriculture sector), and Pneumonia detection from X-ray Images (Image Classification/medical field).

**Results:**

The CNN implementation was done and a comprehensive comparison was done on the platforms to identify the performance, throughput, bottlenecks, and training time. The CNN layer-wise execution in GPU and TPU is explained with layer-wise analysis. The impact of the fully connected layer and convolutional layer on the network is analyzed. The challenges faced during the acceleration process were discussed and future works are identified.

## Introduction

The deep learning field has been widely used in image processing and classification (Huang et al., 2017a), the medical field (Anaya-Isaza, Mera-Jiménez & Zequera-Diaz, 2021; Kikkisetti et al., 2020), speech recognition (Nurvitadhi et al., 2017), and natural language processing and translations (Amodei et al., 2016; Egger et al., 2021). With the rapid growth in data and model size, there is a need for better and robust hardware and software resources like the packages and most advanced libraries for data processing and the faster training of complex models. For image processing applications, the deep neural network (DNN) used widely is Convolutional Neural Network (CNN). CNN works based on the visual system, which was first depicted in Wu et al. (2016), where the visual cortex of a cat was studied, and it was found the receptive field is a sub-region in the visual field which is highly sensitive to visual cortex cells and they detect the light in the receptive fields. Neocognitron (Hubel & Wiesel, 1962) was the first computer-simulated model of the visual cortex, and it was considered as the first step to the development of CNN, which was based on the relation among the neurons for the image transformations. A multi-layer artificial neural network-based CNN framework was put forward by Fukushima (1980), Lecun et al. (1998a) and Lecun et al. (1998a) known as LeNet-5. The LeNet-5 was trained based on the backpropagation algorithm (Lecun et al., 1998a) for the classification of the MNIST dataset for digit recognition from the raw pixels directly without the feature extraction steps. LeNet-5 was a robust algorithm used in many complex applications like document recognition but accuracy was limited due to the lack of computational power and data, it failed to have better accuracy in complex problems (Lecun et al., 1998b). With the use of GPU in machine learning (Bengio et al., 2007), the models started to perform well, and better energy efficient methods were introduced (Krizhevsky, Sutskever & Hinton, 2012; Szegedy et al., 2015; Simonyan & Zisserman, 2015). AlexNet (Zeiler & Fergus, 2014)] is the first CNN model to have a significant impact on image processing tasks. Later many networks were introduced with a more significant number of layers and nodes ZFNet (Zeiler & Fergus, 2014), VGGNet, ResNet (Körez & Barişçi, 2020), and GoogleNet (He et al., 2016) for better performance. CNN is more powerful and has a better performance compared to other traditional deep learning algorithms due to automatic feature extraction capability. The main drawback of CNN is the long training time and the complex neural architecture (Hashemi et al., 2016; Kim et al., 2017). The complex neural networks structures take weeks and months to complete the training process in the case of big datasets. The training time of CNN is increased mainly to increase network depth and network parameters. The training of complex CNN needs high computation power and is time-consuming due to a large number of forwarding and backward iterations. CNN computations are inherently parallel and have a large number of floating-point operations like vector operations.

The computations can speed up using high-performance computing devices like GPU, TPU, FPGA, ASIC, MCU accelerators, particular processors, *etc.* The CNN can be easily implemented in the GPU using the general-purpose GPU programming because of the large number of floating-point computations, and the data transfer rate is significantly less in each iteration of the training process. GPUs have a high computational power compared to the CPU at a low cost due to the parallel architecture. GPUs perform parallel computations by a large number of ALU deployed in a single processor. GPUs are very efficient in performing matrix multiplication tasks which is the foremost step in deep learning applications. TPU is an application-specific integrated circuit (ASIC) developed by Google for accelerating machine learning algorithms and deep neural networks. TPU is cheaper compared to GPU and is available on the cloud. TPU has a good performance and better speed due to the tensors used for computation.

The paper is organized as the related study is done is given in section 2. In section 3, the Methodology is discussed with details of CNN, Implementation in GPU, Implementation in TPU. In section 4, the implementation details are discussed. In section 5, the results are analyzed, and inferences are noted. In section 6, the future scope is identified, and the paper is concluded.

The novel CNN implementation was done, and a comprehensive comparison was made on the platforms to identify the performance, throughput, bottlenecks, and training time. The cross-platform comparison is made for CNN in GPU, TPU, and CPU. The significant contributions in this paper provide a layer-wise execution and analysis of CNN in GPU and TPU. The impact of the fully connected layer and convolutional layer on the network is analyzed. The challenges faced during the acceleration process were discussed, and future directions were identified. The designing of CNN based on the task being MISD (Multiple Instruction Single Data) tasks to make it more effective in TPU. The standard pre-trained network VGG16 and ResNet was also compared with the novel CNN model for finding the impact on training time and accuracy. The accuracy was found to increase for pre-trained models compared to the CNN model used in this work, and this is clearly due to a large number of trainable layers and the pretraining done in the network, but this is achieved by compromising the training time.

### Related Study

Performance and Scalability of GPU on the Convolution Neural Networks (Khan et al., 2020) were implemented on a framework for accelerating training and classification of arbitrary Convolution Neural Networks (CNNs) on the GPU. CNN is a particular case of MLP neural net, and the computation task of CNN runs efficiently in GPU. Based on the CNN topology, classification and training on GPU is two to 24 times faster than CPU (Strigl, Kofler & Podlipnig, 2010). In object detection in RCNN with low-capacity GPU systems (Nurvitadhi et al., 2017), here the object detection plays a vital role in the present technology like agricultural and traffic management, city and town planning, *etc.* A new and faster version of CNN like R-CNN was proposed for GPU. In this model, batch normalization was replaced with weight standardization to make it more efficient for small batches. In white blood cell classification using CNN in GPU and CPU (Sze et al., 2017), the task of finding the white blood cells is not easy. It was implemented in Python using the Keras framework, and the performance was compared in CPU and GPU, and GPU performance was found to be better than CPU.

The studies have been conducted for neural network performance in different platforms. The software tools were analyzed and evaluated in many datasets by Shi et al. (2016) in single and multiple GPU. The CNN algorithm was studied and evaluated in different DL frameworks by Kim et al. (2017) with optimization methods. In the study, the application and different convolutional algorithms were studied for achieving better performance in CNN. The memory usage of deep neural networks in GPU was studied, and the virtualization method was suggested by Rhu et al. (2016). Data reuse was suggested for memory management. The existing works just evaluate the performance in GPU, but in this work, the performance and training time is analyzed with impact on network structure in TPU and GPU.

The deep neural network architectures were evaluated in the heterogeneous systems (Nvidia Titan X and Jenson TX1) (Bianco et al., 2018). In the analysis, the computational and model complexity, accuracy, and memory usage, and training time were shown. From the analysis, the relation between the model complexity and memory usage was clearly shown. The model with low complexity could not effectively run on the test system setup due to the high GPU memory usage. The deep neural network applications were executed using Open CL and CUDA for conducting the study on the latest GPU platforms (Server class GPU, Mobile GPU, and mobile FPGA) (Karki et al., 2019). From the analysis, it was concluded the mobile FPGA is power efficient. The benchmark suite was implemented for many deep neural network applications like speech recognition, machine translation, and image processing (Zhu et al., 2018). The performance analysis was alone done focusing on the memory usage by the neural networks. The neural networks implemented in GPU - (NIVIDA Titan x) and CPU (-Intel Xenon) and the speed and accuracy were compared (Huang et al., 2017b). The majority of the literature discusses the performance, computational cost, memory usage, and training time alone, and the layer-wise analysis and its impact on accuracy and training time are not made.

### Methodology

### CNN: Convolution neural networks

**Figure 1:**
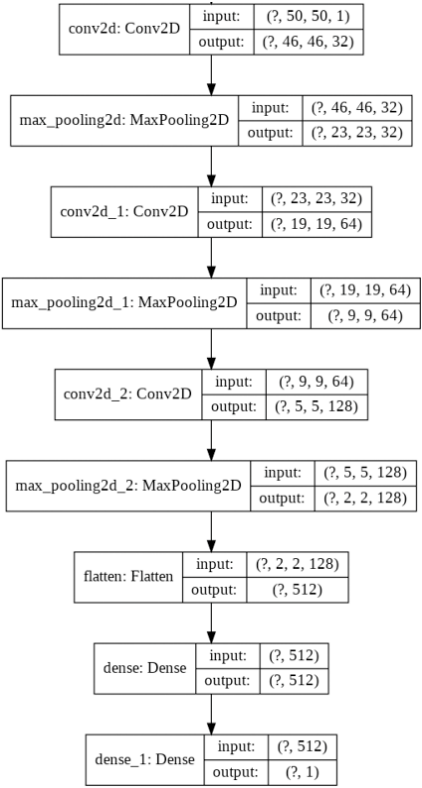
Proposed CNN structure for binary classification.

**Figure 2:**
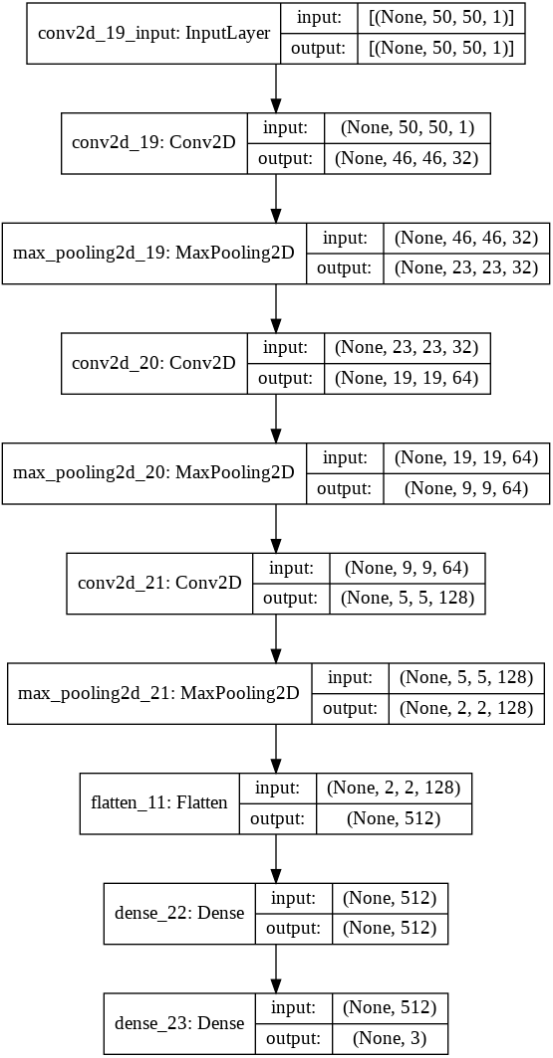
Proposed CNN structure for multiclassifier.

Convolutional Neural Networks contain convolutional layers, pooling layers, striding, activation function, dropout, and fully connected layers. CNN is a combination of Weight sharing, Local Receptive Fields, and temporal, spatial subsampling. The CNN structure is closely related to the computing methods, and so the acceleration is achieved when the steps are implemented. The first convolutional layers are composed of kernels (Körez & Barişçi, 2020) for feature extraction, and each neuron acts as a kernel. The kernels are multiped with the weights, and the results are obtained, and it extracts the feature map from the input images given. Padding can be done for image adjustment to the kernel size. Striding is done to avoid overfitting. In the pooling layer, the information is summed up in the neighborhood and the most prominent feature in that region (Khan et al., 2020). Pooling is performed as a downsampling method. Striding is done to avoid overfitting in the network. The non-linear nature is added to the features using the activation function. The nonlinearity is approximated using the activation layer. The Convolution and pooling layer is the linear process of accumulation of features. Activation functions mainly used are the ReLU, Leaky ReLU, sigmoid, max out, MISH, tanh, Softmax, SWISH, which helps to perfect the approximation of the CNN. Batch normalization is done to solve the slow convergence problem. The feature map is unified using batch normalization by changing the men to zero and variance to unit value (Nurvitadhi et al., 2017). The classification is done at the end by introducing the fully connected layer. The input from the previous layers is fed to the fully connected layer, which gives the classification output based on the previous layer features extracted (Sze et al., 2017). The CNN architecture needs high computing hardware, complicated data traffic routes, and high memory capacity, which makes the implementation very difficult. CNN architecture has evolved during the past few years highly, and detailed studies of them have been done (Ravikumar & Harini, 2020). The CNN performance can be improved if the modules are split and parallelly implemented as different parts. Pruning can also be deployed for cutting the connections. But the pruning and splitting of the network can lead to performance degradation. CNN operations can be split mainly into convolution, pooling, flattening, and fully connected neural network layer (Classification). Both the operations are a sequence of multiplication and addition performed on input and weights. The neurons (multiplication and summation operation) are the main building blocks of a fully connected layer, and it is a matrix–vector operation. Convolution is a GEMM operation (matrix multiplication). The Activation function introduces the nonlinearity in the CNN model. The CNN model proposed has three convolutional layers and followed by the max-pooling layer, flatten layer with the ReLU activation function, and the final fully connected layer. The fully connected layer of CNN is MAC (Multiply and Accumulate) operation which can also be parallelized effectively using temporal and spatial parallel computing architecture like TPU and GPU. In parallel temporal architecture, the vectors and threads (SIMD /SIMT) can be utilized in GPU. The parameter optimization and the neural network structure design has been done for many applications like cryptanalysis (Liu et al., 2022) The neural network structure based on the number of hidden layers and the final classification layer have an impact on the CNN performance, and it is studied in both TPU and GPU platforms. The two CNN network is designed to vary the classes in the final classification layer. The first CNN is for binary classification, as shown in Fig. 1. and the multiclassifier is shown in Fig. 2.

### GPU based acceleration of CNN

GPU is widely used in neural network applications due to a large number of ALU units which helps in faster data processing (multiplication and summation operations in NN), and also the GPU caches, which help in data reuse. The GPU is capable of merging the multiple data access requests using the controllers, and it helps in massive parallel and pipelined processing. This helps to achieve high performance and throughput than CPU for the same DL applications. GPU architecture aims mainly for high instruction throughput and not to reduce the latency in a single instruction. GPU cores are more compared to CPU, and multiple threads run in parallel in CPU. GPU is a temporal architecture paradigm with a large number of ALUs, but the ALUs lack direct data communication, and they communicate using direct memory access.

**Figure 3:**
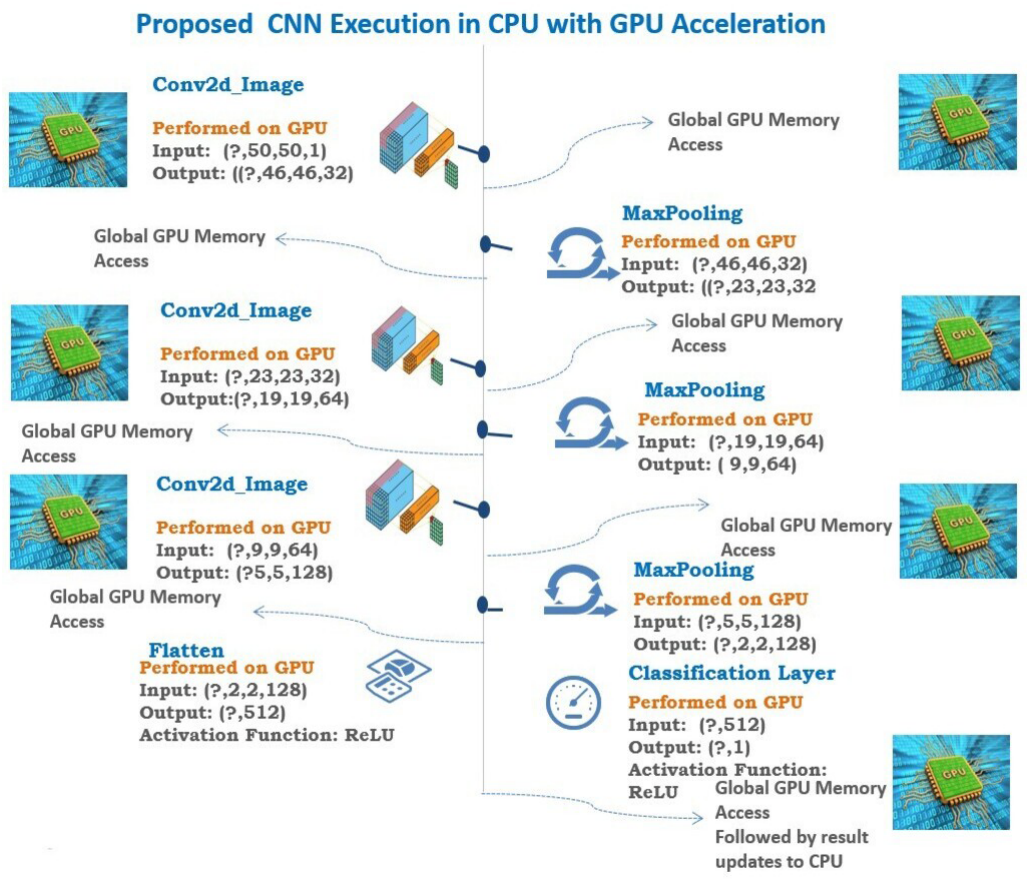
Proposed CNN execution in CPU with GPU acceleration.

The GPU has around 3,000–5,000 ALU inside a single processor, which performs a large number of addition and multiplication operations parallelly. But the Von Neumann bottleneck exists in GPU due to the access to registers and the shared memory for intermediate data storage in every ALU operation. During each operation, the ALU fetch data and store its memory, and ALUs cannot communicate with each other directly. All this leads to memory traffic. All this makes GPU high energy consumption, memory requirement, power-intensive and complex wiring which finally leads to a reduction in the throughput. In the field of computer vision, GPUs have been a significant breakthrough by providing the faster and parallel computation capability needed by the convolutional neural networks (Wu et al., 2016). In CNN, general matrix multiplications of floating (GEMM)-point data are used, which can be effectively processed parallelly in GPU (Krizhevsky, Sutskever & Hinton, 2012). The GPU has specialized libraries for CNN acceleration like fbfft (Vasilache et al., 2015) and cuDNN (Chetlur et al., 2014). While using a high working set, the shared memory cannot be used, and there is a need for global memory access in GPU, and this leads to more memory footprints and memory access. The SIMD/SIMT architecture of GPU is a reason for high DRAM access. In CNN, the convolution, pooling, flatten, and classification layer is executed, and the final result is passed onto the CPU. In GPUs, the throughput is increased using the computational transformation function on the kernels in CNN. The execution of each layer in CNN in GPU is clearly shown in Fig. 3.

### TPU based acceleration of CNN

TPU is a custom-made ASIC with a matrix processor which is specially designed for neural networks. TPU effectively handles the addition and multiplication in neural nets at a very high speed with very little power consumption. The von Neumann bottleneck in CPU and GPU is overcome in TPU with the systolic array structure. TPU v2 single processor has 16 bit two 128 × 128 systolic arrays with 32,768 ALUs. In TPU, the parameters are loaded into the multipliers and adders, and later the data is loaded from memory. The multiplication is performed, and it is propagated to the next multiplier, and summation is performed in parallel. In TPU the memory access is not needed in this process of parallel computations, which helps to achieve high computational throughput and lower the power consumptions on neural networks. The TPU helps to accelerate the GEMM - general matrix multiplications of floating-point data, which is the central part of CNN (Krizhevsky, Sutskever & Hinton, 2012). Systolic array in TPU helps in data reuse which makes the performance high and execution energy efficient in CNN. TPU has a spatial architectural structure and works on dataflow processing in which ALUs communicate with each other directly and even have a local memory/scratchpad. In this data, reuse is done to reduce the consumption of energy and memory access. The numerical explanation of the TPU of NVIDA was done and floating point operation was studied and its shortcomings was identified and non-monotonicity issue that concern the floating point was explained (Fasi et al., 2021).

**Figure 4:**
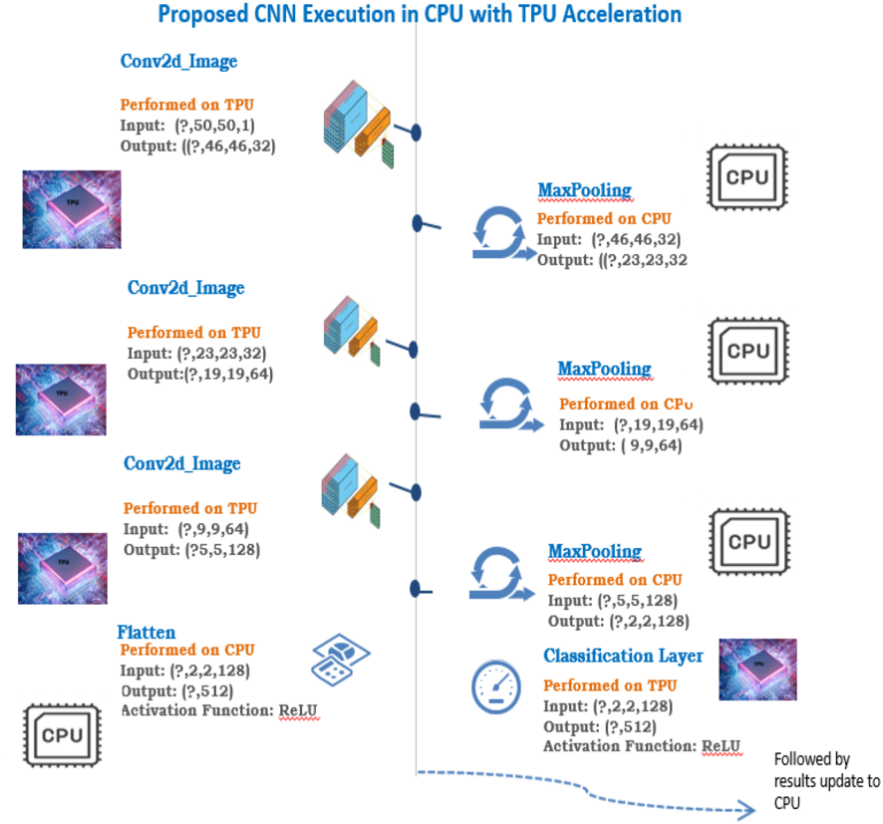
Proposed CNN execution in CPU with TPU acceleration.

The main factors that increase the energy and performance efficiency in TPU are the single processor in TPU, which fixes the latency within a limit compared to multi-threaded CPU and GPU. The two-dimensional multiply unit helps in matrix multiplication faster compared to the one-dimensional multiply units in CPU and GPU. The Systolic arrays help in making the process faster with reduced memory access. In TPU, eight-bit integers are used in place of the 32-bit floating-point operations, and this makes the computations faster and memory efficient. Unlike CPU and GPU, TPU drops features that are not used in the neural network, which helps in saving energy. CNN implementation in TPU will have both TPU and CPU usage in parallel to run the linear and non-linear elements in CNN. In CNN, the convolution and classification layer is executed in TPU since it is a GEMM operation, and the Pooling and Flattening are executed in the CPU. The execution of each layer in CNN in GPU is clearly shown in Fig. 4.

### Implementation

**Figure 5:**
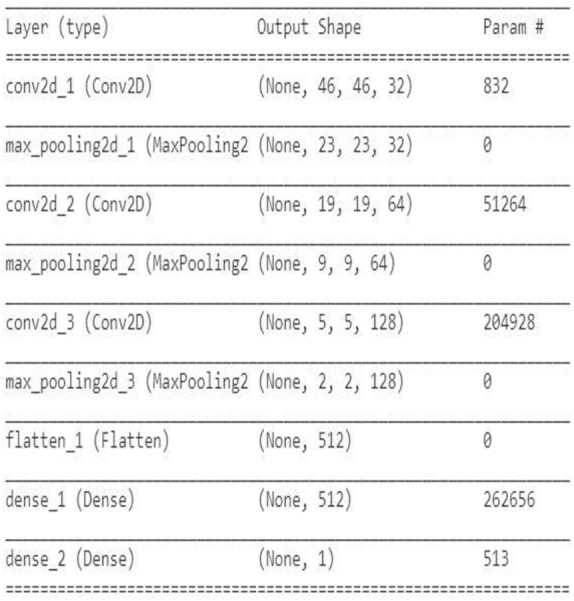
Proposed CNN structure for binary class.

**Figure 6:**
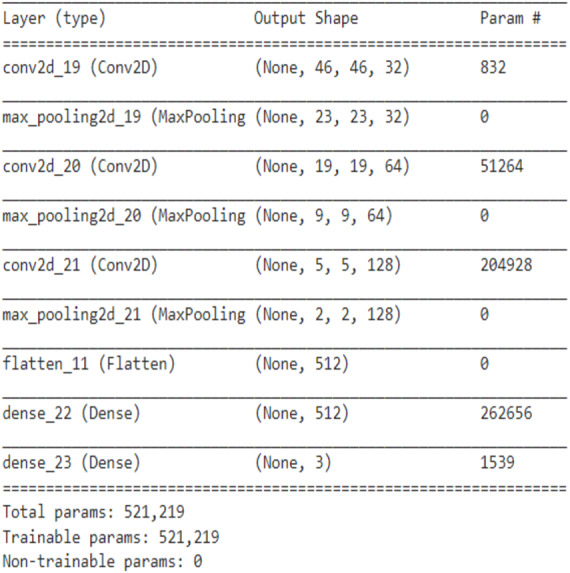
Proposed CNN structure in multiclass classification.

The CNN network design was implemented in Google Colab using the Python Programming language. Google hosted Colab for the AL, ML, Deep learning applications with many inbuilt libraries and free GPU, TPU accelerators. The libraries Keras, TensorFlow, NumPy, pandas, OpenCV, sci-kit learn matplotlib is used. TensorFlow 2.0 is with Keras embedded with the function of. Keras. The CNN architecture used in this work is three sets of Convolution layers, and max-pooling layers followed by a flatten layer, one hidden layer, and one output layer with binary and multiclass classification is shown in Figs. 5 and 6. The input shape given to the model is 50*50*1, so the images need to be resized into 50*50 and need to convert into grayscale images. In the first convolution layer, there are 32 filters, in the 2nd 64 filters, and in the 3rd 128 filters. The hidden layer contains 512 nodes, and the output contains one node, which has a value of 1/0. The dataset for three different computer vision applications is taken, and the dataset (Gurav, 2022; Mooney, 2022) is divided as 80% for training, 10% for validation, and 10% for testing. The data shuffling is applied before splitting is done. The CNN network was designed after trial and error, and the network was designed to avoid both Overfitting and underfitting.

In CPU-based implementation in colab, the image was read, converted into a grayscale image, and resized to 50*50. CPU in Google colab Intel^®^ Xeon^®^, 2.30 GHz CPU Frequency, 2 CPU Core,12GB RAM, and 25 GB disk space. Inbuilt library OpenCV is used for these preprocessing steps. Then the image is converted into a NumPy array, added to the training dataset along with the one-hot encoded label. To train the dataset using the Pytorch framework, initially, it is converted into o a tensor using “torch. Tensor()”. For execution GPU in colab, the initial CPU code can be converted to Cuda program using (Cuda) functions. It runs the code in GPU and distributes the work among all the processors. Initially, all the variables and classes are set to CPU, so to configure the for GPU use to(device) to the class or variable. The GPU available in goggle colab is used Nvidia K80/T4 with 12 GB/16 GB memory, 0.82 GHz/1.58 GHz GPU Memory, 4.1 TFLOPS/8.1 TFLOPS Performance, 2 CPU cores, 12 GB RAM, 358 GB Disk Space.

In TPU, the parameters are sent from memory into matrix adders, and matrix multipliers load the data which is from memory. As multiplication is executed, the output in each multiplier moves to the next multiplier. The result would be the summation of all the multiplied results of parameters. During this process of massive calculations and data passing, memory access is not required, so that is why high computational throughput on neural networks can be achieved by TPU. In Colab, TPU addressing is done using gRPC (gRPCis a modern, open-source remote procedure call (RPC) framework that can run anywhere. It enables client and server applications to communicate and makes it to build connected systems). TPU Cluster Resolver does help to bring the TPU address and creates a cluster to work on; the resolver is used to create the initializing system. Even in TPU, devices are created but are not converted as it is done GPU. It uses a distributed strategy called TPU strategy, and we pass a resolver to TPU strategy, and strategy is the final output. The strategy is like devices created in GPU for working in TPU. The variables created within the strategy scope will be replicated all across the replicas while using distributed strategies. experimental_connect_to_cluster will make devices on the cluster available to use, *i.e.,* calling this more of them will work but will invalidate any tensors which are on old remotes devices. Initialize_tpu_system(tpu) helps to initialize the device. Colab TPU contains eight cores, so our training data is distributed among eight cores which speeds up the process. The model is saved as an instance using Keras Callbacks which can later be used for testing accuracies. But to compare the time differences, we are not stopping the model till it reaches 30 epochs. Three benchmark application the face mask detection, pneumonia detection and the plant disease were taken for this work. These applications are chosen considering the main real-life applications and medical sector. In the current covid situation the face mask detection is a socially relevant application (Kumar et al., 2021), the pneumonia detection is a case from the medical field and disease detection (Elwahsh et al., 2021) and the plant disease detection is from the agricultural sector.

### Face mask detection

The facemask dataset (Gurav, 2022) contains 5,045 training images of people with mask images 2,485, without mask images 1,828 for binary classification. For multiclass classification, 2,485 training images of people with mask images, people without mask images 1,828, and 732 mask images. The CNN model was trained using the facemask dataset. In the face mask application, the live video is implemented in which the live video is captured from the camera, and then it is separated into frames and then the frames compared with the models as created before to find whether the mask is there or not. The video is divided into frames where we pre-process the frame and send it to the saved model to predict with mask/without a mask. A rectangular box is drawn over the face and displays whether it is a mask or no mask along with accuracy. The input dataset needs to be pre-processed to decrease the complexity and to make the dataset fit in the neural network. Pre-processing steps used are the conversion of the image into the greyscale image, resizing the image into 50*50, which is the input shape given to the neural network—converting the image into NumPya array and rescaling the array values to 0 to 255 by dividing each pixel by 255. There will be no lag in detecting the face mask from the video as we are using high-performance distributed systems. This model helps to analyze how much people are aware of wearing the mask in different places through those specific actions will be taken by the government on them for increasing the awareness in people. The application was implemented in the same CNN model, varying the final fully connected layer. In the first case, it was done as a binary classifier that identifies the person with and without mask using a ReLU activation function in the final output layer. The second analysis was done varying the final activation function to SoftMax for multiclass prediction.

### Leaf disease identification

Leaf dataset (Emmanuel, 2022) in which dataset contains 2,000 images, in which 1,000 are early bright tomato leaf images and 1,000 tomato old leaf images for binary classification. The multiclassifier dataset contains 3,591 images, of which 1,591 are healthy leaf images, and 1000 are early bright tomato leaf images, and 1000 tomato old leaf images. Agriculture in India is a livelihood for a majority of the population, wherein in 2020, 41.49 percent of the workforce in India were employed in agriculture. Major crops grown in India are rice, wheat, millets, pulses, tea, coffee, and jute, *etc.* An estimated 15–25 percent of potential crop production is lost due to plant diseases. Manual searches and getting solutions to plant diseases are quite difficult for farmers. Even if the disease is identified, they should know the solutions to overcome the disease. Therefore, we need an application that helps the farmers to detect the disease in the early stages. The application was implemented in the same CNN model, varying the final fully connected layer. In the first case, it was done as a binary classifier that identifies the infected and healthy leaf using a ReLU activation function in the final output layer. The second analysis was done varying the final activation function to SoftMax for multiclass prediction.

### Pneumonia detection

Pneumonia X-ray dataset (Mooney, 2022) with 1,586 images in which 846 are pneumonia level 1 affected images and 740 are pneumonia level 2 affected images for binary classification. The multiclassifier dataset contains 2,927 images, of which 1,341 healthy images, 846 are pneumonia level 1 affected images, and 740 are pneumonia level 2 affected images. Pneumonia is a respiratory infection, and that affects the lungs. The alveoli in the lungs will be infected, which makes breathing painful and limits oxygen intake. There are around 5 million people in India who are suffering from pneumonia, and India records an average of 4,000 deaths every year. We use chest X-rays to diagnose the infection, and we need expert doctors and radiotherapists to check the infection from the X-rays.

## Results

The CNN model performance was analyzed for the three-image processing application in GPU/TPU platforms in Colab for various batch sizes. The analysis was done varying the final feed-forward network and the hidden layers, and this gives an inference on how the performance is affected when the model structure changes. In the CNN model chosen, the input data supplied for the three processors remains the same, so the batch size is varied to compare the performance. The training time, accuracy of the model in GPU and TPU is analyzed.

**Figure 7:**
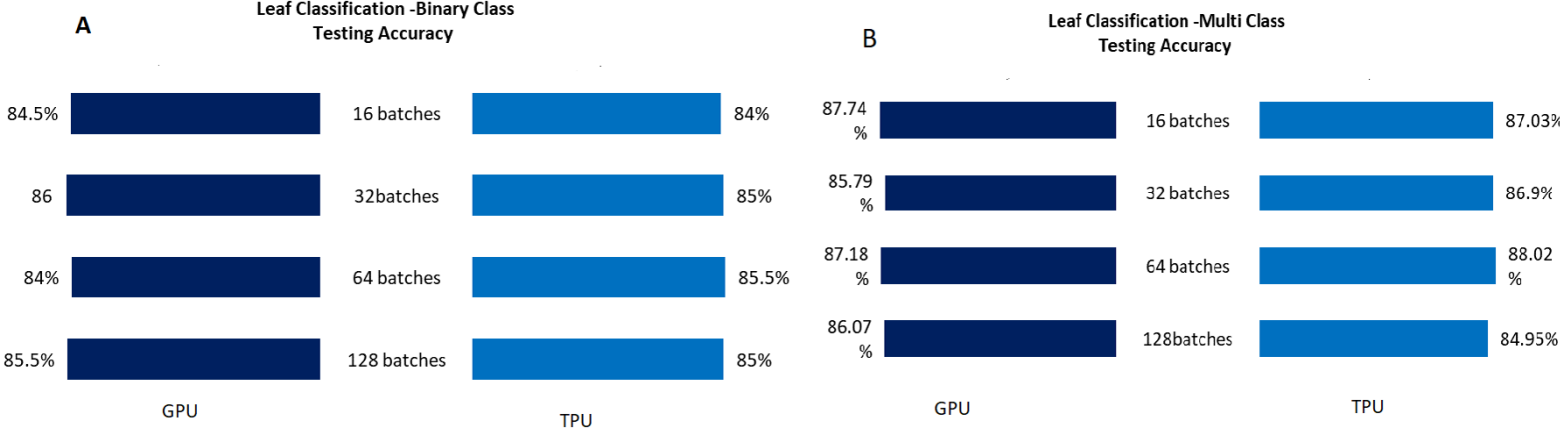
Leaf classification-binary and multiclass testing accuracy. (A) Leaf classification testing accuracy for binary class. (B) Leaf classification testing accuracy for multiclass.

**Figure 8:**
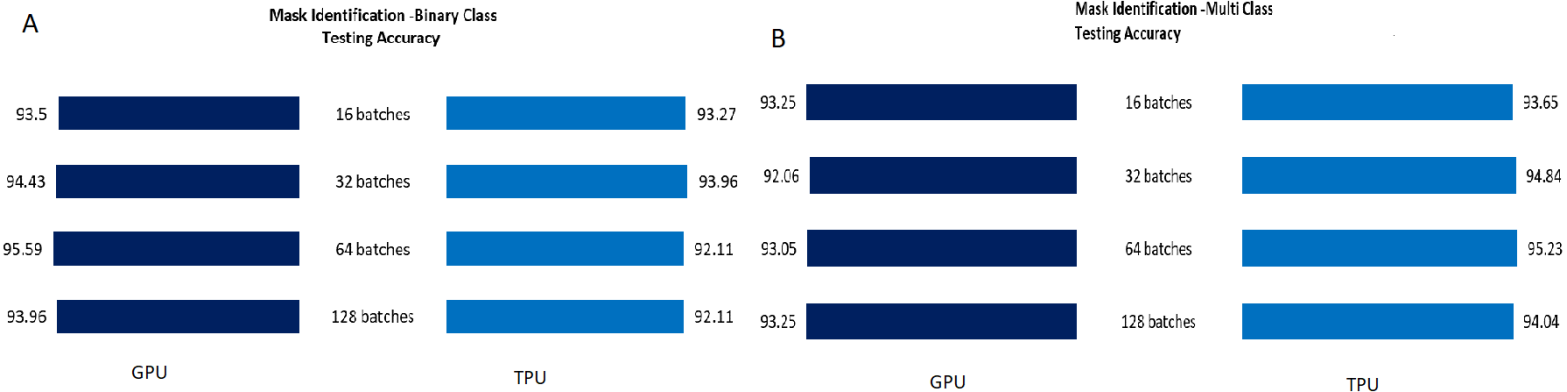
Mask classification-binary and multiclass testing accuracy. (A) Mask classification testing accuracy for binary class. (B) Mask classification testing accuracy for multiclass.

**Figure 9:**
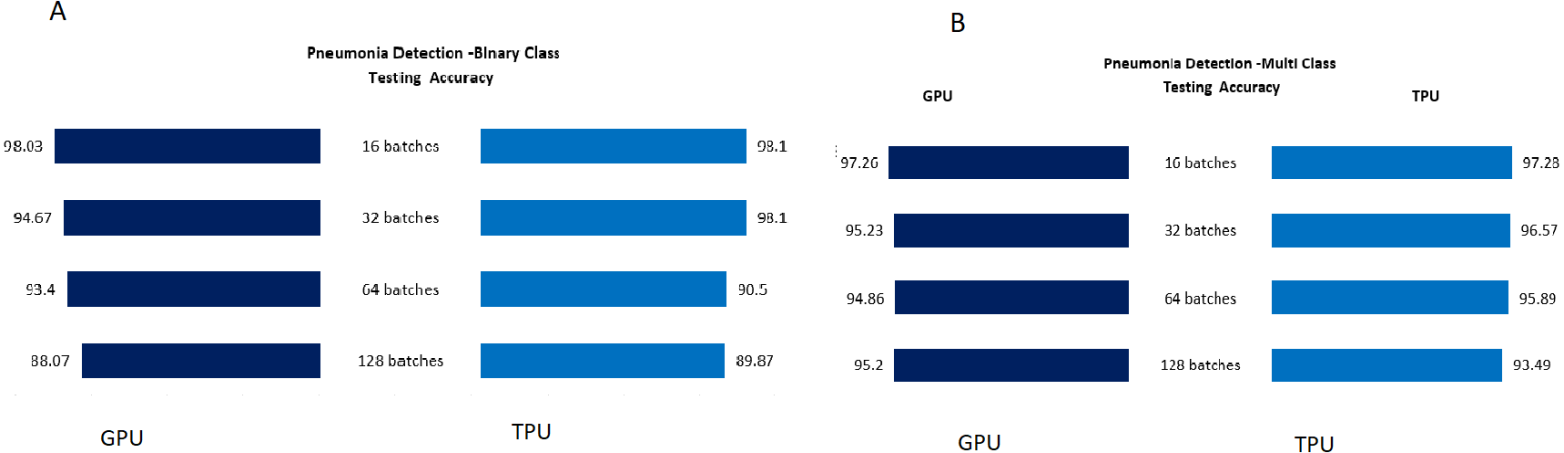
Pneumonia detection: binary and multiclass testing accuracy. (A) Pneumonia classification testing accuracy for binary class. (B) Pneumonia classification testing accuracy for multiclass.

The Testing accuracy for each application was compared for both GPU and TPU for batch sizes 16,32,64,128. The accuracy of the leaf disease identification for the binary and multiclassifier is shown in Fig. 7. In Fig. 7, the binary class and multiclass CNN for the leaf dataset were analyzed based on accuracy, and it shows that the accuracy of the multiclass increases compared to binary class for both GPU and TPU. The accuracy of the mask identification for the binary and multiclassifier is shown in Fig. 8. In Fig. 8, binary class and multiclass CNN for the mask dataset were analyzed based on accuracy, and it shows that the accuracy of the multiclass increases compared to binary class for both GPU and TPU. The accuracy of the pneumonia detection for the binary and multiclassifier is shown in Fig. 9. In Fig. 9, the binary class and multiclass CNN for the Pneumonia Detection dataset were analyzed based on accuracy, and it shows that the accuracy of the multiclass increases compared to binary class for both GPU and TPU. From the analysis, it was clear that the accuracy remains almost the same in GPU and TPU for both multiple and binary classifiers, and this shows that accuracy is not affected by the parallelization process.

**Figure 10:**
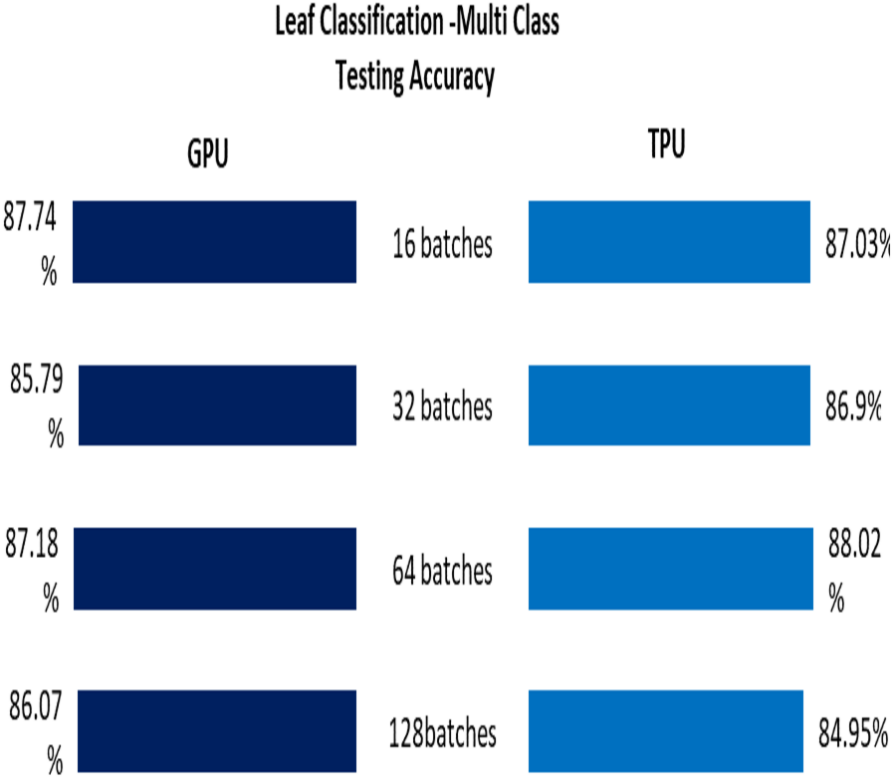
CNN structure with single convolutional layer.

**Figure 11:**
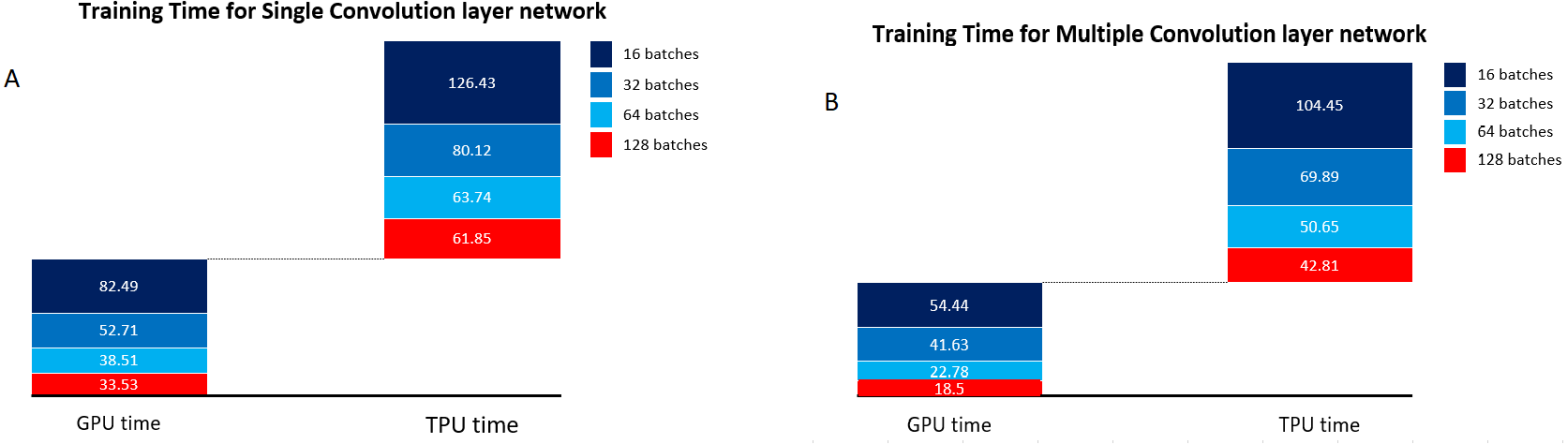
Training time for single and multiple convolutional layer network. (A) Training time for single convolutional layer network. (B) Training time for multiple convolutional layer network.

The analysis was done using a single convolutional layer followed by all other layers for the mask detection application for binary class, and the training time was analyzed for GPU and TPU for both networks. The training time increases when the convolutional layers are removed because the number of nodes gets more, and thereby training time increases. The single convolutional layer CNN architecture is shown in Fig. 10. The training time for Single layer convolution and multiple-layer Convolution for different batch sizes is shown in Fig. 11. The analysis clearly shows that the time decreases when the number of convolutions increases due to a reduction in the number of nodes. The training time is less for the multiple layers CNN compared to single-layer CNN and also with an increase in the batch size.

**Figure 12:**
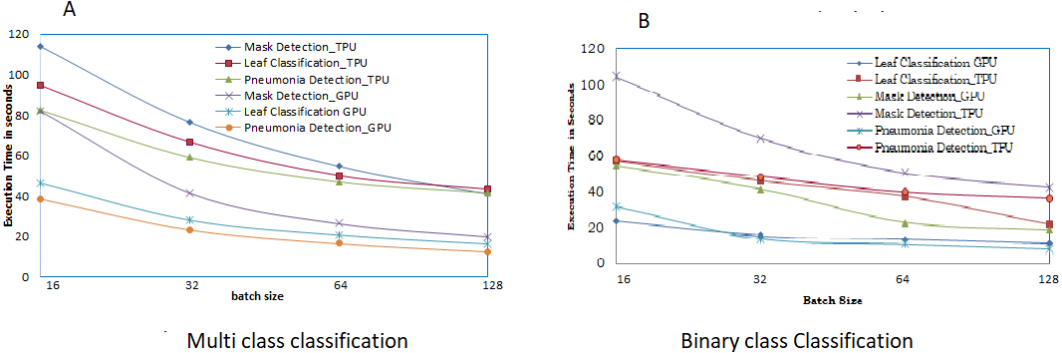
Execution time for multi and binary class. (A) Training time of multiclass classification for benchmark applications. (B) Training time of binary-class classification for benchmark applications.

The overall training time for each of the three applications was in GPU and TPU for both binary, and multiple classifications were analyzed and shown in Fig. 12. From Fig. 12, it is clear that compared to TPU, GPU has a low time for execution of the CNN. This occurs due to the bottleneck that occurs in TPU due to the in-between CPU access.

**Figure 13:**
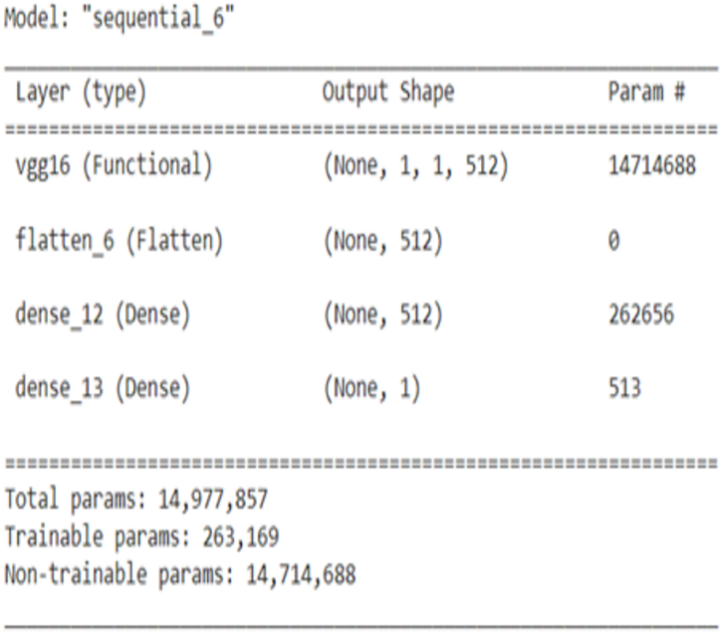
VGG16 CNN structure.

**Figure 14:**
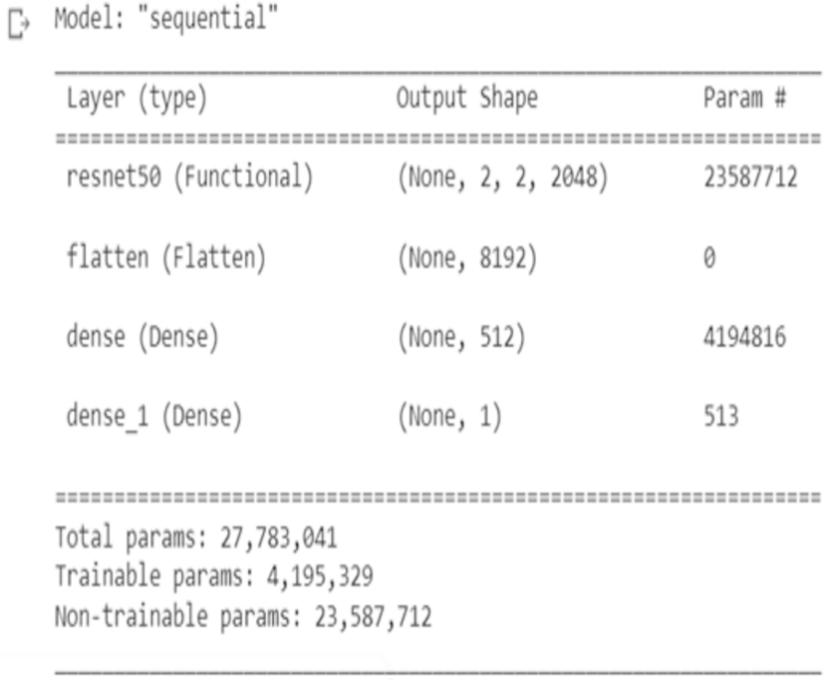
ResNet CNN struture.

**Figure 15:**
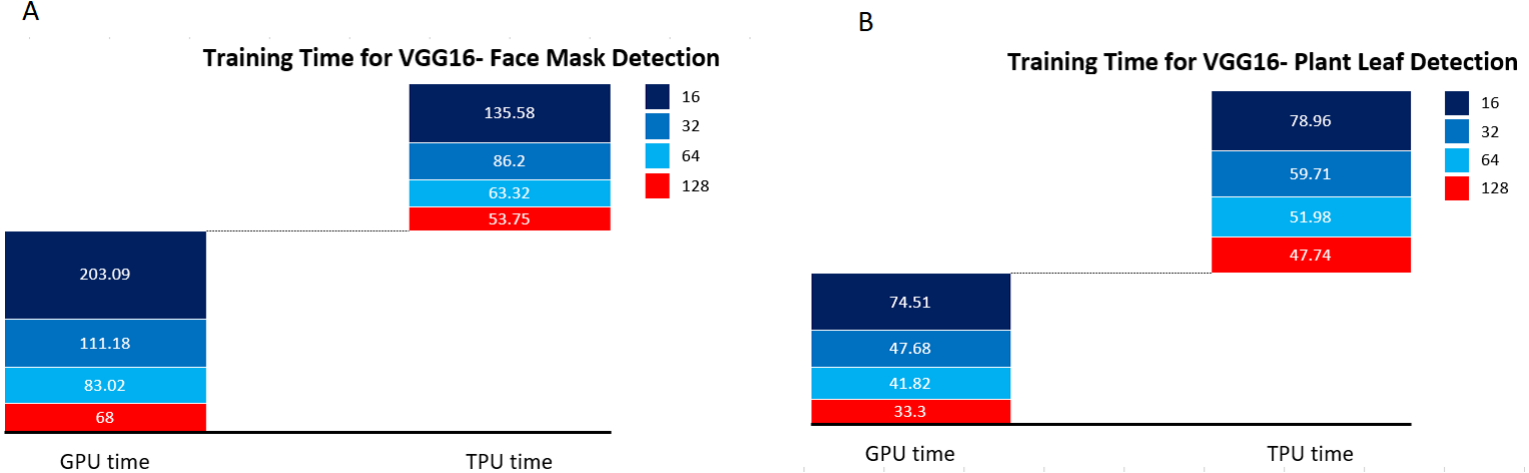
VGG16 training time. (A) Training time for VGG16- face mask detection. (B) Training time for VGG16- plant leaf detection.

**Figure 16:**
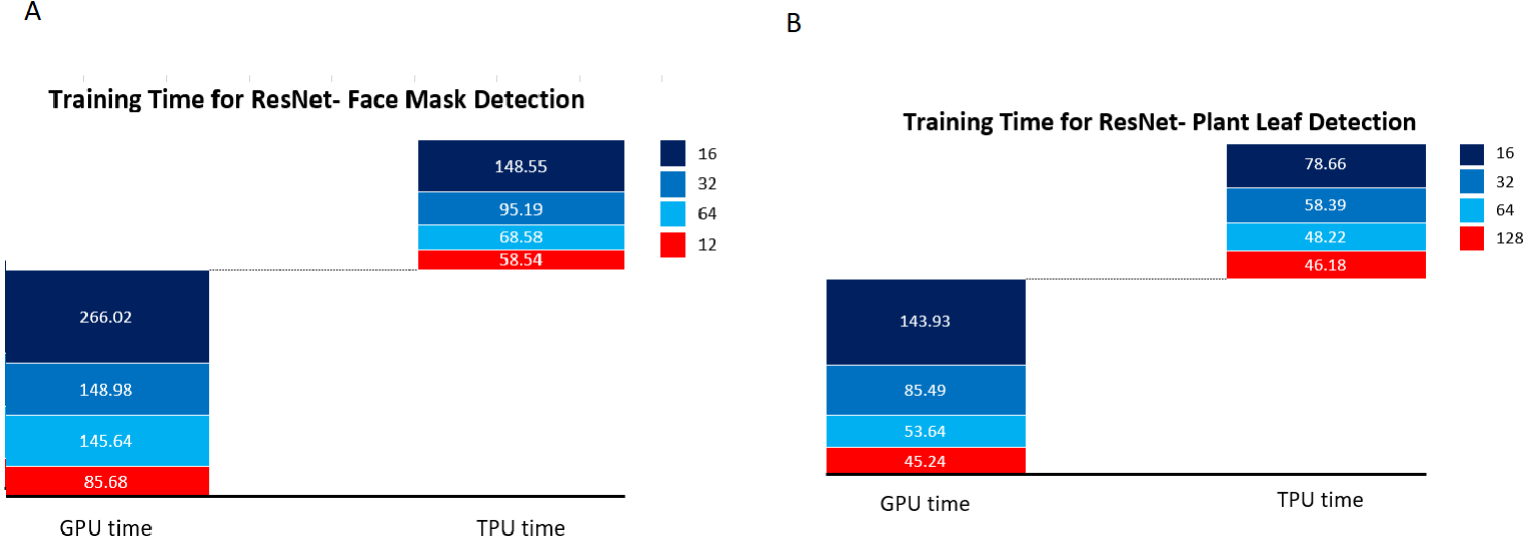
ResNet training time. (A) Training time for ResNet face mask detection. (B) Training time for ResNet plant leaf etection.

The Plant leaf and mask dataset was applied for the standard pre-trained network VGG16 and ResNet, and the network structure for VGG16 is shown in Fig. 13 and ResNet in Fig. 14. The principle of transfer learning was used for the training of the network in which the pre-trained network weights were taken. The network was executed for the Plant leaf dataset and the face mask dataset. VGG 16 network was executed for batches 16, 32, 64, and 128, and the single-layer convolutional model was used. The VGG 16 was found to take more training time compared to our network due to the time taken to load the trained network. From Fig. 15, it is clear that the training time of the VGG network is more compared to the designed network. ResNet network was executed for batches 16,32,64, and 128, and the single-layer convolutional model was used. The ResNet was found to take more training time compared to our network due to the time taken to load the trained network. From Fig. 16, it is clear that the training time of ResNet is more compared to the designed network. The accuracy was found to increase for pre-trained models compared to the CNN model used in this work, and this is clearly due to a large number of trainable layers and the pretraining done in the network, but this is achieved by compromising the training time.

## Discussion

Cross-Platform analysis was done using CPU, GPU, and TPU on the same novel CNN. From the analysis, the key takeaways are:

• The Global GPU memory access is a problem faced in GPU implementation of CNN, but in the latest GPU versions of GE Force by NVIDIA with the Turning Microarchitecture, the Shared memory can be utilized, and this problem can be removed.
• The CNN implementation in GPU and TPU was analyzed layer-wise, and the places where the bottleneck occurs in TPU and GPU were identified. The Convolutional network should be designed with each task being MISD (Multiple Instruction Single Data) tasks to make it more effective in TPU. The neural network tasks must be given importance while designing a network.
• GPU: GPU performs well for small batches and gives better flexibility and easy programming. For small data, batch sizes GPU fits better due to the execution pattern in wraps and scheduling id easy on-stream multiprocessors. For large dataset and network models, GPU performs well by optimizing memory reuse. In fully connected neural networks, weight reuse is less, so as the model size increases, this leads to high memory traffic. In GPU, the memory bandwidth makes it practical for applications with memory requirements. Large neural networks work better on GPU compared to CPU due to the extra parallelism feature. For fully connected neural networks, GPU works better compared to CPU, but for large batch sizes, TPU performs well.
• TPU: TPU performs well on CNN with large batches to give high throughput in training time using the systolic array structure. Large batches of data are needed for the full utilization of the matrix multiply units in the systolic array of TPU. In CNN, the speedup increases with batch size. For enormous batch sizes and complex CNN, TPU is the best because of the spatial reuse characteristics of CNNs. But in fully connected networks, the weight reuse is less, and so TPU is not preferred.

## Conclusions and Future Work

Deep learning has been growing at an exponential rate in the last few years due to its wide real-world applications. The accuracy of DNN depends on the computing power, the parameter size, and the network complexity. The complex DNN needs high computational requirements, which cannot be handled by a standard CPU, so there is a need for hardware accelerators. The GPU and TPU accelerators are studied and their effect on CNN architecture. GPU is effective for DNN with the large number of ALU, but the considerable memory access due to the single instruction multiple data architecture causes a problem. In complex Convolutional Neural Networks with high DRAM access and a large number of floating-point computations, the GPU is not sufficient. The specialized neural network-based ASIC accelerators for tensor computations (TPU) are used. TPU performs well on CNN with large batches to give high throughput in training time using the systolic array structure. For large batch sizes and complex CNN, TPU is the best because of the spatial reuse characteristics of CNNs. But in fully connected networks, the weight reuse is less, and so TPU is not preferred. CNN structure can be mainly split as convolution, pooling, and fully connected network. Each part has different computation requirements, and how they are executed in GPU and TPU is clearly explained in work. In the future, the CNN structure can be designed to use the capability of TPU effectively. As a future extension of the work, the impact of convolutional and pooling layers needs to be analyzed depth-wise, and the network design must be done based on the task and its impact on the training time and performance. The work can be extended to multi-node clusters. As max pooling reduces the parameters and takes the max value from matrix and shorten the parameters. Max pooling is used to detect the exact features even if the image is rotated or shirked. In max pooling layer we will take 2 × 2 or 4 × 4 matrix from the image and note the max number from the matrix so that the high-level feature will be marked even the image is rotated and we are reducing the size by 75 percent and it helps to prevent the overfitting by removing the extra information. The drop out layers can be incorporated with the CNN which helps in reducing the overfitting of data and will lead to better output.

This work guides the selection of the most appropriate platform for CNN implementation and gives insight into how the fully connected layer of CNN affects the output. The performance, training time of the platforms were analyzed for three different image applications for a novel CNN. No particular platform is suitable for all scenarios. The training time, memory, and energy usage have to be considered before fixing a platform. The detailed analysis of performance in terms of training time and accuracy is done for both GPU/TPU /CPU for sample image applications, and the bottlenecks are explained. A complete understanding of the CNN algorithm used, the dataset, batch size, and hardware is required for the selection of the appropriate accelerator for an application. The main two understandings that we concluded from this study is the direct connectivity ratio (ratio of the number of layers that are directly connected) to the total layers and indirect connectivity (ratio of number of layers that are transitively connected) of CNN is a major factor that determine the network performance.

## Additional Information and Declarations

### Data Availability

The following information was supplied regarding data availability: The code is available in GitHub: https://github.com/Maruthi-Saketh-2001/Analysis-of-deep-learning-applications-in-cpu-gpu-tpu. The data is available at Kaggle: - https://www.kaggle.com/omkargurav/face-mask-dataset
- https://www.kaggle.com/emmarex/plantdisease
- https://www.kaggle.com/paultimothymooney/chest-xray-pneumonia.
